# Ultrasound-based assessment of muscle mass is associated with early recovery after kidney transplant: a prospective single-center study

**DOI:** 10.1186/s12871-025-03288-4

**Published:** 2025-08-26

**Authors:** Nicholas V. Mendez, Michael P. Bokoch, Dieter Adelmann, Matthew D. Bucknor, Elaine Ku, Kerstin Kolodzie

**Affiliations:** 1 Department of Anesthesia & Perioperative Care, University of California, San Francisco, San Francisco, CA 94143 USA; 2 Department of Radiology & Biomedical Imaging, University of California, San Francisco, San Francisco, CA 94143 USA; 3 Department of Medicine, Division of Nephrology, University of California, San Francisco, San Francisco, CA 94143 USA; 4 Department of Epidemiology & Biostatistics, University of California, San Francisco, San Francisco, CA 94143 USA; 5 Philip R. Lee Institute for Health Policy Studies, University of California, San Francisco, San Francisco, CA 94143 USA; 6 Department of Anesthesia and Perioperative Care, University of California, San Francisco, 521 Parnassus Avenue #4210, San Francisco, CA 94117 USA

**Keywords:** Kidney transplant, Muscle mass, Ultrasound, Postoperative outcomes

## Abstract

**Background:**

Low muscle mass and frailty are associated with worse perioperative outcomes. However, traditional modalities for quantifying muscle mass are limited, costly, may require radiation exposure, and can be unreliable with kidney failure. We hypothesize that ultrasound-measured muscle mass is associated with early recovery metrics after kidney transplant.

**Methods:**

In a prospective single center cohort study, we investigated the association between muscle mass and short-term outcomes after kidney transplant. Patients undergoing kidney transplant between November 2019 and October 2020 were enrolled. We quantified muscle mass by ultrasound measurement of the rectus femoris cross-sectional area. The primary outcome was the number of days alive and out of hospital within 30 days of surgery. Incidence of surgical complications by the Clavien-Dindo system was also evaluated.

**Results:**

Thirty-eight patients were enrolled with 36 completing kidney transplant. Median cross-sectional area was 4.82cm^2^ [IQR 4.18 to 6.05] and median days alive and out of hospital was 26 [IQR 24 to 27]. Lower muscle mass was associated fewer days alive and out of hospital postoperatively. Cross-sectional area was 4.35cm^2^ [IQR 4.11 to 5.79] versus 5.49cm^2^ [IQR 4.94 to 6.55] for those at or below versus above the median days alive and out of hospital respectively (p = 0.046). Lower muscle mass was associated with occurrence of at least one surgical complication. Cross-sectional area was 4.30cm^2^ [IQR 4.11 to 4.91] versus 5.46cm^2^ [IQR 4.35 to 6.84] for those who did and did not experience a complication respectively (p = 0.024).

**Conclusions:**

Lower muscle mass as measured by point-of-care ultrasound was associated with fewer days alive and out of hospital and more surgical complications after kidney transplant. Further studies should explore the role that ultrasound-measured muscle mass can play in guiding the pre-surgical care of patients prior to kidney transplant.

**Supplementary Information:**

The online version contains supplementary material available at 10.1186/s12871-025-03288-4.

## Background

Kidney failure is a rapidly growing global health concern with over 850 million people estimated to be living with kidney disease worldwide [1]. Preexisting comorbidities and poor functional status among patients with kidney failure are linked with early postoperative complications after kidney transplant, which are costly and associated with increased morbidity and mortality [2]. The ability to identify patients at high-risk of early postoperative complications is clinically important for designing interventions such as prehabilitation programs. Such programs would enable optimization of transplant candidates while awaiting transplant, thus both improving patient outcomes and facilitating optimal use of scarce donor organ resources [3].

Decreased skeletal muscle mass, one component of frailty and a surrogate for poor functional status, has been associated with perioperative adverse outcomes including an increased risk of prolonged hospitalization, readmission, and mortality across various surgical populations when measured by conventional tools [4]. Muscle mass is commonly quantified by computed tomography (CT) of the psoas muscle, dual-energy x-ray absorptiometry, or bioelectrical impedance analysis. These technologies are resource-intensive, limited in availability, can require radiation exposure, and may be costly [5–7]. Furthermore, kidney failure and dialysis are both known to accelerate protein catabolism, which is physiologically distinct from age-related muscle loss [8]. Therefore, the applicability of these conventional imaging modalities in quantifying muscle mass are sometimes questioned in the kidney transplant population [8, 9].

Bedside ultrasound has emerged as a tool to evaluate muscle mass, correlating with frailty and adverse postoperative outcomes in the non-transplant population [10–12]. For example, rectus femoris (RF) cross-sectional area (CSA) measured by ultrasound has been associated with prolonged hospitalization and the need for discharge to a skilled nursing facility (SNF) in surgical intensive care unit (ICU) patients [11]. Additionally, it has been demonstrated that ultrasound-measured RF CSA can reliably measure muscle mass in patients with chronic kidney disease and end-stage renal disease [13–15]. In this study, we evaluated RF CSA by point-of-care ultrasound as a reproducible, non-invasive bedside technique for assessing the risk of adverse postoperative outcomes in kidney transplant patients. We hypothesized that a lower muscle mass preoperatively is associated with poorer patient-centered early recovery metrics postoperatively.

## Methods

Ethical approval was obtained from the University of California, San Francisco Institutional Review Board, protocol #19–28382 in October 2019 and written informed consent was obtained from all participants. We conducted a prospective single center observational cohort study investigating the association between preoperative muscle mass and short-term recovery metrics following deceased-donor kidney transplant. Patients aged 18 years or greater were recruited between November 2019 and October 2020 when presenting for kidney transplant at a large academic medical center, which performs over 300 kidney transplants annually. Patients were recruited 24 h per day, such that patient enrollment was not dependent on the timing of the transplant. Enrollment paused between March 2020 and June 2020 due to COVID-19-related on-site work restrictions for research staff. Patients were excluded if they were non-English speaking, intellectually disabled, prisoners, multiorgan transplant recipients, or if they self-reported a bilateral lower extremity (LE) disability. The cohort was limited to deceased-donor organ recipients as living-donor organ recipients oftentimes spend less time on dialysis preoperatively, have fewer comorbidities, fewer postoperative complications, and the donor organ often experiences a shorter cold ischemia time [16]. A session of dialysis is not routinely performed prior to transplant unless otherwise clinically indicated. Patients undergoing kidney transplant at our center present to the hospital from home prior to surgery and are transferred to the post-anesthesia care unit (recovery room) postoperatively without the need for ICU admission unless otherwise clinically indicated. All patients underwent deceased-donor kidney transplant per the intraoperative protocol described previously [17]. Patients receive induction immunosuppression after induction of general anesthesia with methylprednisolone 500 mg followed by either basiliximab or anti-thymocyte globulin at the discretion of the kidney transplant service. All immunosuppressive agents were administered after the muscle mass measurements occurred. We followed the Strengthening the Reporting of Observational Studies in Epidemiology guidelines in preparation of this manuscript [18].

### Ultrasound measurements

**Fig. 1:**
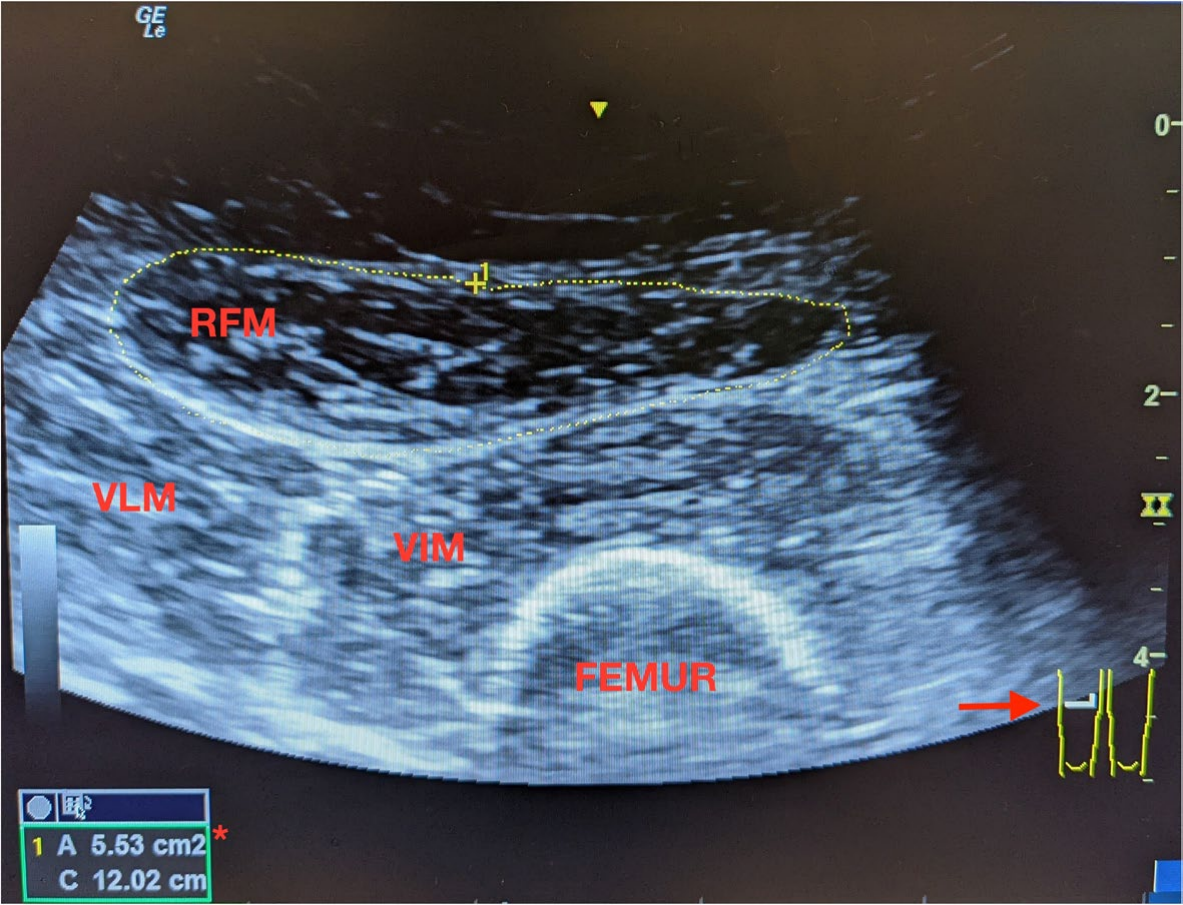
An example of the ultrasound images acquired for measurement of the rectus femoris cross-sectional area using a wide curvilinear-array transducer. RFM: rectus femoris muscle; VIM: vastus intermedius muscle; VLM: vastus lateralis muscle; Arrow: demonstrates probe location on right thigh during imaging

One faculty anesthesiologist experienced in ultrasound measured all RF CSAs in centimeters squared (cm^2^) using a GE Logiq™ *e* (Illinois, USA) and wide curvilinear-array transducer. Patients were positioned supine with head-of-bed at 45 degrees and legs passively extended. Transverse images of the RF muscle were acquired with the probe perpendicular to the point representing 66% of the distance of the anatomic line connecting the anterior superior iliac spine to the upper pole of the patella. The inner echogenic line of the RF muscle was traced manually on a frozen image at bedside to calculate the RF CSA as described by Mueller et al. [11] A sample image from a patient in the study is presented (Fig. 1). The first ultrasound measurement was performed in the preoperative holding area within approximately the one hour prior to transplant. Reproducibility was assessed by replicate measurement of the RF CSA of same laterality, performed by the same operator at least 10 min after the first image with both images being acquired before proceeding to the operating room for kidney transplant. If dialysis was clinically indicated before transplant, the ultrasound measurements were performed after the dialysis session. The stronger extremity was imaged if the patient self-reported a one-sided LE disability. The mean of the first and repeat measurements for each patient was calculated and considered the final value.

### Primary outcome

The primary outcome was days alive and out of hospital within 30 days (DAH_30_) post-kidney transplant, which is validated as a patient-centered outcome in perioperative clinical studies [19, 20]. DAH_30_ was calculated using hospital length of stay (LOS), readmission, and mortality data from the time of kidney transplant (day 0) until postoperative day (POD) 30 [20]. Described by Myles et al., DAH_30_ reflects the efficacy and efficiency of perioperative care, integrating factors important to patients including postoperative LOS, readmission, and early death after surgery. DAH_30_ inherently aligns with a patient’s desire to recover quickly, minimize complications, and forego readmission [21].

### Secondary outcomes

Secondary outcomes included occurrence of surgical complications and patient-reported quality of recovery postoperatively, both collected before accessing and analyzing study data. Postoperative surgical complications were assessed using the Clavien-Dindo classification system at time of discharge [22]. Clavien-Dindo classification grades surgical complication severity on the type of therapy needed to correct the complication and has been endorsed by several medical professional societies including The Transplantation Society [22–25]. The Clavien-Dindo classification consists of five grades of increasing severity: grade I, any deviation from the standard postoperative course without need for a pharmacologic or procedural intervention; grade II, requiring pharmacological treatment, blood transfusion, or total parenteral nutrition; grade IIIa, requiring procedural intervention not under general anesthesia; grade IIIb, requiring procedural intervention under general anesthesia; grade IVa, single organ dysfunction (including dialysis); grade IVb, multi-organ dysfunction; and grade 5, patient demise [22]. Delayed graft function (DGF) was not considered a surgical complication.

Patient-reported quality of recovery after surgery was evaluated using the quality of recovery 15-item (QoR-15) survey. QoR-15 is a patient-centered outcome validated for evaluating a patient’s early postoperative health status in perioperative clinical studies [26]. The QoR-15 short-form questionnaire was administered by a trained interviewer at baseline (before surgery), POD 3, and POD 30. The survey includes 15 questions, each rated on an 11-point scale (0 to 10), creating a sum score between 0 and 150 (0 for poor recovery through 150 for excellent recovery).

### Sample size

Considering that our primary outcome likely would not be normally distributed and no linear association between RF CSA and DAH_30_ could be established, we estimated the sample size needed if the outcome variable was categorized into a binary variable [11]. We chose to dichotomize the variable DAH_30_ at the median as there is no universally accepted number of DAH_30_ that is considered a favorable outcome for kidney transplant patients in the literature. Furthermore, we considered a difference of 2 cm^2^ with a standard deviation (SD) of 2 as clinically meaningful based on studies in the non-transplant population [11, 21]. This difference of 2 cm^2^ was intentionally selected as a conservative choice for our population given the considerably higher prevalence of sarcopenia in patients with kidney failure and therefore lower expected variability of muscle mass. With α = 0.05 (two-sided) and β = 0.2, a sample size of 34 patients was needed to detect statistical significance. To account for potential losses to follow-up, we planned to recruit 38 patients.

### Statistical analysis

Categorical variables are expressed as counts and percentages and continuous variables as medians with interquartile ranges (IQRs) or means with SDs. As predetermined, the outcome variables will be categorized into binary variables divided at the median. As such, differences in baseline and donor organ characteristics between the groups of patients who experienced greater than the median versus the median or fewer DAH_30_ were calculated using Pearson’s X^2^ test for categorical variables and Wilcoxon rank-sum test for continuous variables. Reproducibility of the first and repeat measurements of RF CSA were assessed by calculating the Intraclass Correlation Coefficient (ICC). ICC is a reliability index widely used to evaluate intrarater and interrater reliability, including in ultrasound-based assessments of muscle mass [10, 27]. Bland–Altman (BA) plot displaying the within subject mean against the within subject SD of the two ultrasound measurements was plotted to confirm constant variance over the range of means. Two-sample Wilcoxon rank-sum test was used to compare distributions of the RF CSA rankings between patients with DAH_30_ above versus at or below the median as well as between patients who did and did not experience a surgical complication. QoR-15 scores over time were displayed in a profile plot for patients with RF CSA at and above the median versus below the median. We fitted a mixed effect model to compare the groups over time to account for the repeated measurement of the QoR-15 scores. *P*-values < 0.05 were considered statistically significant. Statistical analysis was performed using Stata (Version 17.0, StataCorp LLC, USA) and figures created using GraphPad Prism (Version 10.0, GraphPad Software Inc, USA).

## Results

**Fig. 2:**
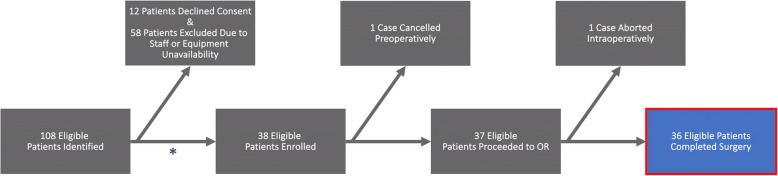
Study flow diagram demonstrating patient enrollment, timepoint of exclusions, and final study population. Initially, 108 eligible patients were identified, of which 12 declined to consent and 58 were excluded due to staff and/or equipment unavailability. In total, 38 patients were enrolled with one case cancelled preoperatively and one case aborted intraoperatively after induction of general anesthesia but prior to incision, resulting in a final cohort of 36 patients. (*) Of note, enrollment of patients was paused between March 2020 and June 2020 due to on-site work restrictions for research staff related to the COVID-19 pandemic

Of 108 eligible patients, 12 declined consent and 58 were excluded due to staff or equipment unavailability resulting from on-site work restrictions during the early months of the COVID-19 pandemic. Thus, 38 patients were enrolled in the study. One case was cancelled after enrollment and after first RF CSA measurement but before the repeat measurement and before surgery due to concerns surrounding inadequacy of presurgical optimization. As such, 37 patients had two RF CSA measurements completed. Another case was cancelled intraoperatively after induction of anesthesia but prior to incision due to patient hemodynamic instability. Overall, 36 patients had both two RF CSA measurements and completed kidney transplant (Fig. 2).

The cohort had a median age of 52 years (IQR 44 to 59), was 56% male, 22% Hispanic, and was racially diverse (22% Asian, 22% Black, 53% White, and 3% Hawaiian/Pacific Islander). Median body mass index was 28 kg/m^2^ (IQR 23 to 33) and median body surface area by the Du Bois method was 1.92 m^2^ (IQR 1.72 to 2.17). While no patients were current smokers, 36% identified as former smokers. All patients were ambulatory and without previous LE amputations, although 8% were limited to level surfaces and one patient self-reported a unilateral LE disability due to hemiplegia after a cerebrovascular accident. Dialysis had not yet been initiated in 11% of the cohort. Median dialysis vintage for those on dialysis was 7.0 years (IQR 3.9 to 9.3), with 75% on hemodialysis and 25% on peritoneal dialysis. The population included 14% of patients undergoing repeat kidney transplant and 19% of organs were from donation after circulatory death. Median kidney donor profile index (KDPI) was 37% (IQR 26 to 58) and 25% of donor organs were placed on machine perfusion prior to transplant. Median RF CSA for the 36 analyzed patients who completed surgery was 4.82 cm^2^ (IQR 4.18 to 6.05).

For the 37 patients with two CSA measurements, the mean of the first and repeat measurements for each patient was calculated and used for all further analyses. The median of the combined RF CSAs was 4.91 cm^2^ (IQR 4.20 to 5.91). The ICC for the first and repeat RF CSAs demonstrated excellent reproducibility with a coefficient of 0.93 (CI 0.89 to 0.98). The BA plot indicated a constant variance of the mean values (see supplemental digital content 1).

**Fig. 3:**
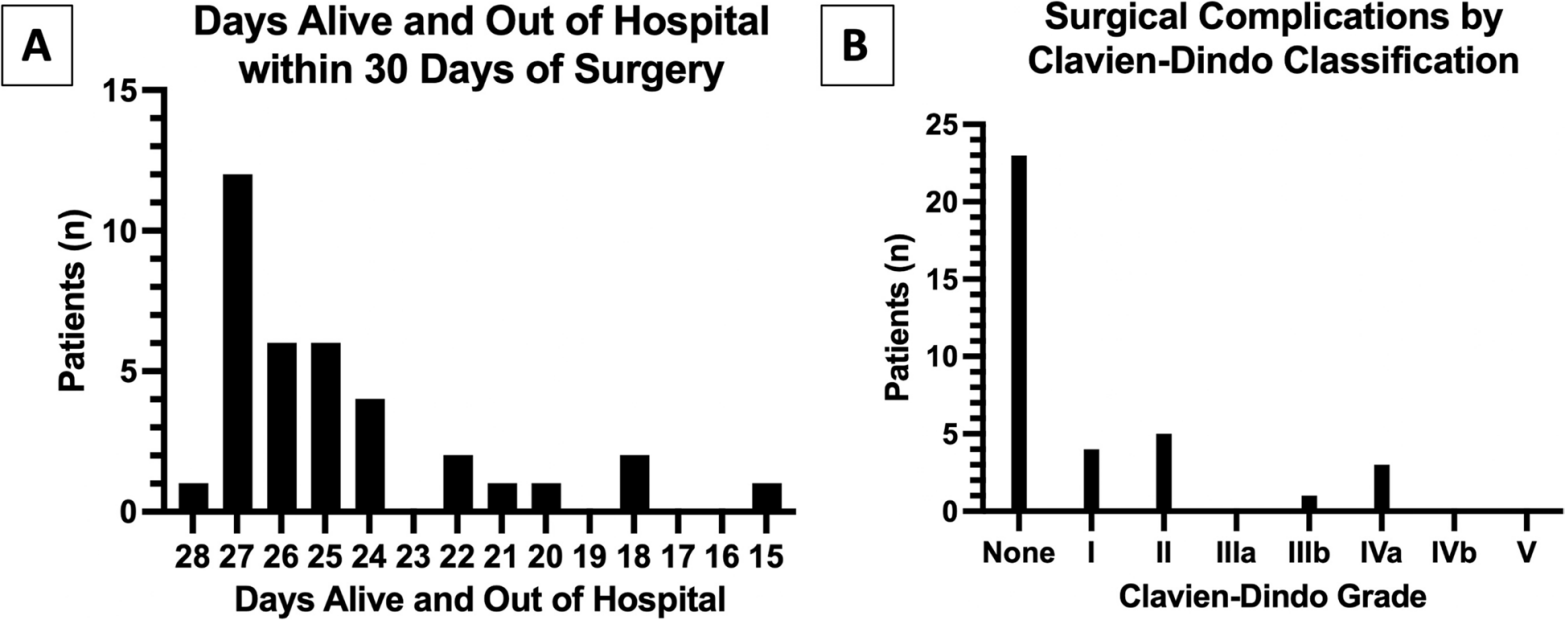
**A** Number of patients who experienced each category of days alive and out of hospital within 30 days of surgery. **B** Number of patients who experienced a postoperative surgical complication distributed by Clavien-Dindo Grade. Grade I: Deviation from the standard postoperative course without the need for a pharmacologic or procedural intervention; Grade II: Pharmacologic treatment initiated; Grade IIIa: Invasive intervention not requiring general anesthesia; Grade IIIb: Invasive intervention requiring general anesthesia; Grade IVa: Single organ failure; Grade IVb: Multi-organ failure; Grade V: Patient demise

**Fig. 4:**
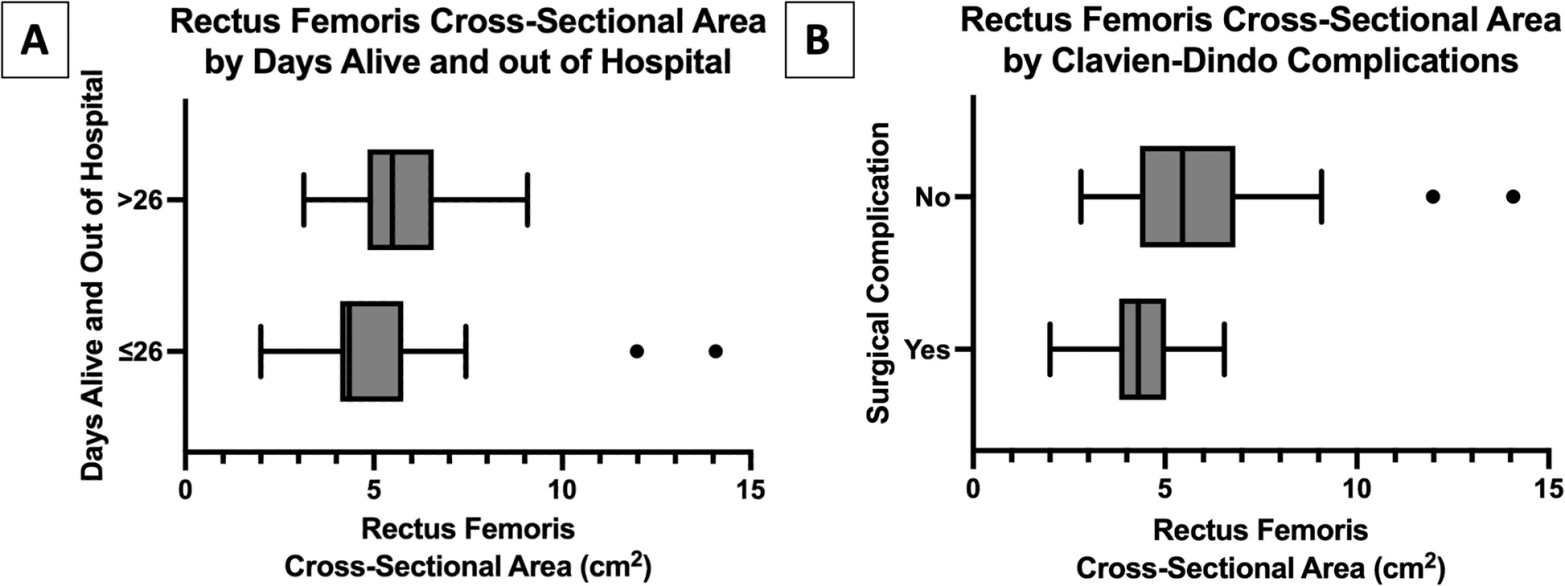
**A** Association between the rectus femoris cross-sectional area measured by ultrasound and the number of days alive and out of hospital within 30 days of surgery (*p* = 0.046). Median of pairwise differences (Hodges-Lehmann estimator): 0.995 (CI95% 0.07 to 1.99) **B** Association between the rectus femoris cross-sectional area measured by ultrasound and the occurrence of at least one postoperative surgical complication by the Clavien-Dindo classification (*p* = 0.024). Median of pairwise differences (Hodges-Lehmann estimator): 1.04 (CI95% 0.11 to 2.23)

Median DAH_30_ was 26 (IQR 24 to 27) with thirteen patients (36%) having the benefit of experiencing more than 26 DAH_30_. One patient (3%) required unanticipated ICU admission postoperatively for continued hemodynamic monitoring due to suspected intraoperative anaphylaxis. Ten patients (28%) experienced at least one hospital readmission. No deaths occurred within the 30-day follow-up period. Since no linear relationship was found between RF CSA and DAH_30_, the primary outcome was dichotomized at the median (26 days) into individuals with greater than 26 versus 26 or fewer DAH_30_ (Fig. 3A). There were no statistically significant differences in baseline or donor organ characteristics between those who experienced greater than 26 versus 26 or fewer DAH_30_ (Table 1). Of note, the difference in KDPI between the two groups was of borderline statistical significance with the group of individuals who experienced 26 or fewer DAH_30_ receiving donor kidneys with a higher KDPI percentage (41 versus 32%, p = 0.054). When comparing RF CSA distribution between the two primary outcome groups, the median CSA for those with more than 26 DAH_30_ was 5.49 cm^2^ (IQR 4.94 to 6.55) compared with 4.35 cm^2^ (IQR 4.11 to 5.79) for those with 26 or fewer DAH_30_ (*p* = 0.046) (Fig. 4A). Using the Hodges-Lehmann estimator, the median of all possible pairwise differences between the two groups was 0.995 (CI95% 0.07 to 1.99). The probability that the RF CSA was larger for any randomly selected patient with more than 26 DAH_30_ compared to a randomly selected patient with 26 or fewer DAH_30_ was 70%. We conducted a sensitivity analysis to assess the robustness as well as to identify influential factors related to this finding, provided in supplemental digital content 2.

**Table 1:** Baseline recipient and donor characteristics

	Total (*n* = 36)	Days Alive and Out of Hospital > Median (*n* = 13)	Days Alive and Out of Hospital ≤ Median (*n* = 23)	*P* Value
**Recipient Characteristics**				
Age, years	52 (44–59)	48 (41–54)	54 (46–62)	0.083
Male	20 (56%)	8 (62%)	12 (52%)	0.587
Hispanic	8 (22%)	4 (31%)	4 (17%)	0.354
Race				
Asian	8 (22%)	3 (23%)	5 (22%)	0.530
Black	8 (22%)	2 (15%)	6 (26%)	
White	19 (53%)	7 (54%)	12 (52%)	
Hawaiian/Pacific Islander	1 (3%)	1 (8%)	0 (0%)	
Height, cm	170 (159–183)	172 (158–180)	168 (160–185)	0.895
Dry weight, kg	81 (67–98)	85 (74–99)	76 (63–96)	0.449
Body mass index, kg/m^2^	28 (23–33)	29 (24–34)	28 (23–33)	0.383
Body surface area (Du Bois), m^2^	1.92 (1.72–2.17)	1.93 (1.87–2.18)	1.91 (1.66–2.16)	0.610
Baseline Comorbidities				
Hypertension	36 (100%)	13 (100%)	23 (100%)	-
Diabetes	9 (25%)	4 (31%)	5 (22%)	0.548
Coronary Artery Disease	9 (25%)	2 (15%)	7 (30%)	0.317
Former Smoker	13 (36%)	4 (31%)	9 (39%)	0.616
Ambulation Status				
Independent	33 (92%)	13 (100%)	20 (87%)	0.174
Limited to level surfaces	3 (8%)	0 (0%)	3 (13%)	
Preemptive transplant	4 (11%)	1 (8%)	3 (13%)	0.624
Time on dialysis, years	7.0 (3.9–9.3)^a^	6.0 (4.2–7.8)^b^	7.4 (2.7–9.8)^c^	0.533
Type of dialysis				
Hemodialysis	24 (75%)^a^	11 (92%)^b^	13 (65%)^c^	0.092
Peritoneal dialysis	8 (25%)^a^	1 (8%)^b^	7 (35%)^c^	
Repeat transplant	5 (14%)	2 (15%)	3 (13%)	0.855
**Donor Organ Characteristics**				
Donation after Circulatory Death	7 (19%)	1 (8%)	6 (26%)	0.180
Kidney Donor Profile Index, %	37 (26–58)	32 (18–35)	41 (29–73)	0.054
Machine Perfusion	9 (25%)	4 (31%)	5 (22%)	0.548
Cold Ischemia Time, min	790 (492–1137)	750 (414–936)	806 (540–1218)	0.138
Warm Ischemia Time, min	30 (25–36)	30 (25–40)	30 (24–35)	0.779

Values presented as medians with 25th and 75th percentiles, or as numbers (n) and percentages (%)

^a^Total *n* = 32, accounting for four preemptive transplant patients in the total cohort

^b^Total *n* = 12, accounting for one preemptive transplant patient in the group of patients who experienced median number of day alive and out of the hospital

^c^Total *n* = 20, accounting for three preemptive transplant patients in the group of patients who experienced ≤ median number of days alive and out of the hospital

Thirteen patients (36%) experienced a postoperative surgical complication of any grade before initial hospital discharge. Since occurrence of surgical complications of any grade was relatively uncommon, with more than one grade having no observations, this outcome was dichotomized into those with no surgical complications versus those with one or more of any grade (Fig. 3B). Median RF CSA for those with a surgical complication was 4.30 cm^2^ (IQR 4.11 to 4.91) compared with 5.46 cm^2^ (IQR 4.35 to 6.84) for those without (*p* = 0.024) (Fig. 4B). Using the Hodges-Lehmann estimator, the median of all possible pairwise differences between the two groups was 1.04 (CI95% 0.11 to 2.23). While not considered a surgical complication, DGF occurred in 16 patients (44%). The median RF CSA for those who developed DGF was 4.39 cm^2^ (IQR 4.17 to 6.86) compared with 4.92 cm^2^ (IQR 4.20 to 5.88) for those who did not (*p* = 0.824).

**Fig. 5:**
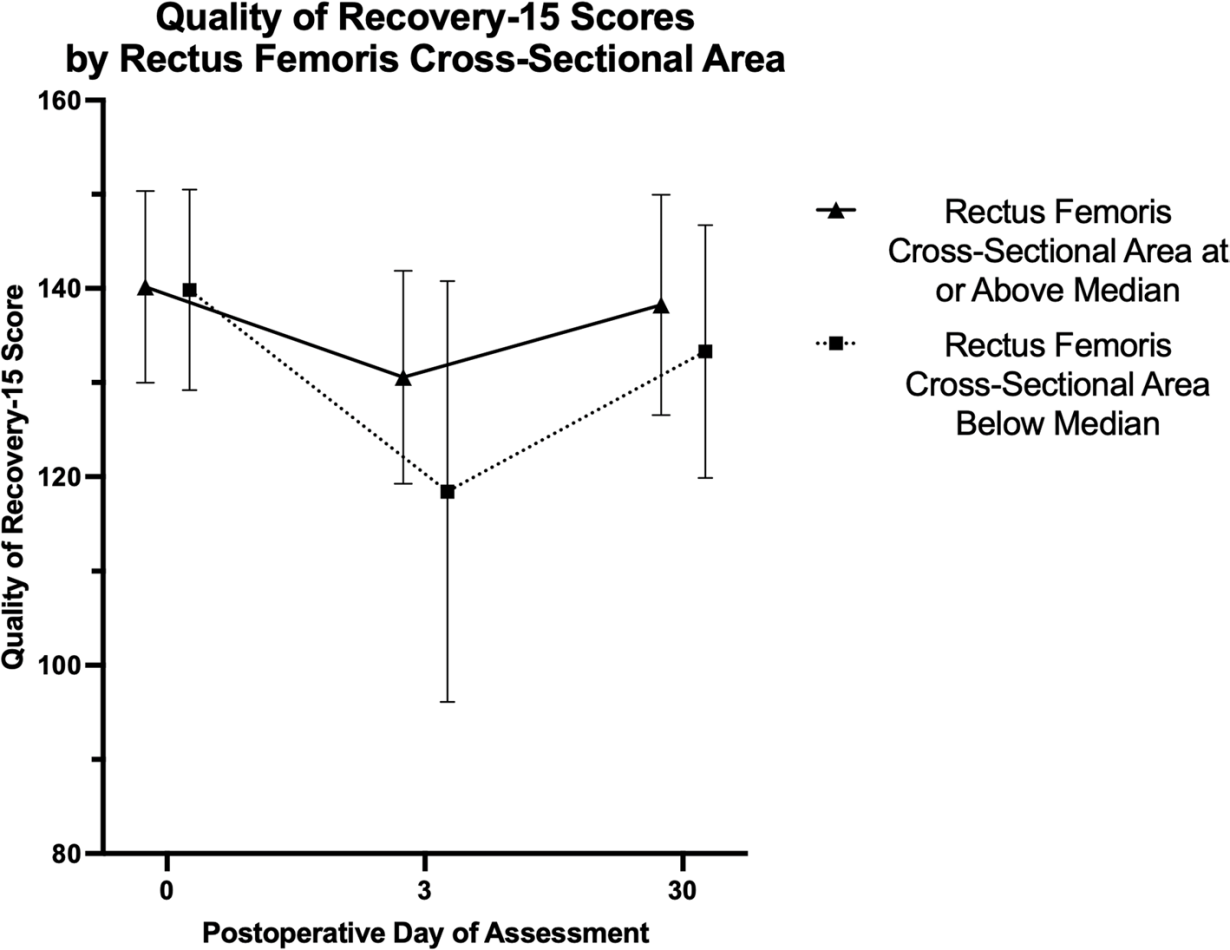
Profile plot summarizing the Quality of Recovery-15 scores for patients with rectus femoris cross-sectional areas at or above the median versus below the median evaluated at baseline (before surgery), postoperative day three, and postoperative day 30. The mean survey scores on postoperative day three for those patients with a muscle mass at or above the median versus below the median was 131 ± 11 versus 118 ± 22 respectively, reflecting a drop in survey score from baseline of 22 points in patients with muscle mass below the median as compared with a drop of only nine points in patients with a preoperative muscle mass above the median

All 36 patients completed the QoR-15 survey at baseline and on POD 3. There was a 92% response rate on POD 30 due to loss to follow-up of three patients. Two patients were discharged and could not be reached by phone, and the third individual was readmitted on POD 30 and declined to complete the questionnaire. Thus, the QoR-15 analysis is based on 36 patients for baseline and POD 3 and 33 patients for POD 30. Mean survey scores at baseline for patients with RF CSA at or above the median was 140 ± 10 versus 140 ± 11 for those below the median. The mean survey scores on POD 3 for patients with RF CSA at or above the median versus those below the median was 131 ± 11 versus 118 ± 22 respectively. The mean survey scores on POD 30 for patients with RF CSA at or above the median versus those below the median was 138 ± 12 versus 133 ± 13 respectively. A profile plot summarizing the QoR-15 survey results at each timepoint is provided (Fig. 5). The mixed effect model comparing QoR-15 scores for patients with RF CSA at and above the median versus below the median over time showed overall a statistically significant difference between groups (*p* = 0.048). At postoperative day 3, the QoR-15 was significantly lower in patients with CSA below the median compared to patients with CSA at or above the median (−12.11 [CI95% −21.19 to −3.03], *p* = 0.009). This is consistent with the clinically significant change in scores of 6 to 8 points or more from baseline as reported by Myles et al. [28, 29] The QoR-15 did not differ at baseline (*p* = 0.898) and at postoperative day 30 (*p* = 0.306), suggesting that the impact of muscle mass on patient’s quality of recovery occurred during the early postoperative period.

## Discussion

We found that measurement of muscle mass by point-of-care ultrasound in kidney transplant patients was highly repeatable. Additionally, we found that a lower preoperative RF CSA by ultrasound was associated with fewer days alive and out of hospital and more surgical complications after kidney transplant. Using RF CSA as a surrogate for muscle mass, we found that lower muscle mass was associated with fewer DAH_30_. Lower muscle mass was also associated with postoperative surgical complications, likely increasing LOS and risk of readmission. When evaluating QoR-15 survey results, patients reported nearly identical QoR-15 scores at baseline regardless of muscle mass but patients with a muscle mass below the median experienced a drop in survey score on POD 3 of 22 points from baseline as compared with a drop of only 9 points in patients with muscle mass above the median. Myles et al. investigated the minimal important difference in QoR-15 scores and reported that a change from baseline of 6 to 8 points or more reflects a clinically important deterioration [28, 29]. This finding aligns with the increased number of postoperative complications in patients with lower muscle mass, further substantiating the argument that a lower preoperative muscle mass is associated with a worse quality of recovery potentially mediated by surgical complications and increased LOS.

Our findings are consistent with reports from the general surgical and ICU populations. Mueller et al. demonstrated that RF CSA by ultrasound is associated with adverse outcomes in surgical ICU patients including unplanned discharge to a SNF, death, and LOS [11]. Furthermore, Canales et al. showed that muscle mass measured by ultrasound is associated with adverse postoperative outcomes including delirium and unplanned SNF admission [12]. Similarly, we observed that a lower muscle mass is associated with fewer DAH_30_. We found that the RF CSA mean difference in our cohort between the two outcome groups of 1.38cm^2^ was already associated with a significant and meaningful difference in outcomes, which is less than the 2cm^2^ difference that was found to be clinically meaningful in a study of a surgical ICU population [11]. This finding is conceivably a reflection of the well-established increased prevalence of sarcopenia in patients with kidney failure and further underscores the importance of investigating this relationship between muscle mass and adverse postoperative outcomes specifically in this unique patient population [8].

In contrast, studies evaluating muscle mass measured by ultrasound and its relationship with adverse perioperative outcomes specifically in kidney transplant patients are limited. More commonly, studies have retrospectively analyzed CT-derived data and reported an association between low preoperative muscle mass and increased LOS, risk of readmission, and risk of mortality [5, 30, 31]. Karakizlis et al. retrospectively analyzed CT-derived data and recently described an association between lower muscle mass and increased LOS as well as graft survival at three years [32]. This time in a prospective cohort, our study corroborates these findings confirming that a lower preoperative muscle mass is associated with worse short-term outcomes after kidney transplant. Our study also provides the benefit of confirming this association by point-of-care ultrasound, which is a noninvasive, low cost, easily repeatable bedside technique.

Obtaining a preoperative CT scan for the sole purpose of evaluating muscle mass is clinically limited by high costs, logistic complexity, and radiation concerns. Necessity and timing of abdominal CT scans prior to kidney transplant vary between centers, limiting the possibility of relying on clinically available scans for frailty evaluation and particularly if implemented in a repeated fashion for tracking over time. In fact, only 14 of the 38 patients in our study had a recent abdominal CT scan available, limiting our ability to perform comparative analyses. Our study offers a new perspective by examining the association between preoperative muscle mass and patient-centered outcomes including DAH_30_ and QoR-15. Lastly, we examined a racially and ethnically diverse cohort, suggesting applicability across varied practice settings and demographics.

Kidney transplant candidates are particularly well positioned to benefit from interventions like preoperative prehabilitation given their oftentimes multiple longstanding comorbidities, relatively long waiting times once qualifying for transplant, and high number of patients removed from the list due to clinical deterioration [33]. While early prehabilitation studies in kidney transplant patients demonstrate promising results, identification of objective, cost effective, and easily repeatable tools for tracking progress over time remains a challenge [3, 34, 35]. With the association between preoperative muscle mass by ultrasound and short-term outcomes established herein, our results lay the foundation for future studies investigating the important role ultrasound can serve in addressing this need.

Despite successfully enrolling the predetermined number of patients, the cohort of this study remains small. This small observational study is intended to be hypothesis-generating and, as such, future studies with larger cohorts are needed to validate these findings as well as to adjust for potentially confounding variables. Future studies may also consider expanding the inclusion criteria to include recipients of living donor organs. Likewise, as a single-center study, the cohort is reflective of the deceased-donor kidney transplant population at our institution, which may not be generalizable to all transplant centers. Additionally, although a high degree of intraobserver reproducibility was observed for the ultrasound measurements, it remains possible that variability may exist when performed by others as all ultrasound images were acquired and measured by the same operator in this study. Future studies should consider acquiring the ultrasound images by multiple operators with varying degrees of experience. These studies may also consider adding a comparator group, examining the comparative reliability and predictive value of ultrasound-measured RF CSA versus other modalities. Future studies could also consider obtaining postoperative muscle mass measurements, as recent evidence suggests that acute kidney pathology, particularly acute kidney injury (AKI), has been linked with muscle wasting [36, 37]. While this study focuses on preoperative muscle mass measurements, this link between AKI and muscle wasting very plausibly has relevance to the kidney transplant population as all donor organs are subjected to some degree of ischemia time and ischemia–reperfusion injury. Future studies can also consider evaluating the possible association between ultrasound-measured RF-CSA and long-term patient outcomes. While this study focuses on short-term perioperative outcomes, it is clinically plausible that preoperative muscle mass, given its apparent association with worse perioperative outcomes, could also be associated with worse long-term outcomes after kidney transplant. Additionally, while we chose to dichotomize the primary outcome (DAH_30_) at the median, future studies with larger cohorts my choose to partition this variable into more segments allowing for overall improved interpretability. Finally, while we encountered some challenges during recruitment due to research staff and/or equipment availability, these were related to the on-site work restrictions imposed during the early months of the COVID-19 pandemic and are unlikely to impact the measurements or outcomes observed for either clinical or research purposes in the future.

## Conclusions

In summary, lower ultrasound-measured preoperative muscle mass is associated with negative postoperative short-term patient-centered outcomes after kidney transplant. Additional studies are needed to explore the role that ultrasound-measured muscle mass can play in guiding the pre-surgical care of patients being considered for kidney transplant.

## Supplementary Information

Supplementary Material 1.

Supplementary Material 2.

## Data Availability

Deidentified data acquired and analyzed during this study are available from the corresponding author on reasonable request.

## Abbreviations

AKI: Acute kidney injury
BA: Bland-Altman
cm^2^: Centimeters squared
CSA: Cross-sectional area
CT: Computed tomography
DAH_30_: Days alive and out of hospital within 30 days
DGF: Delayed graft function
ICC: Intraclass correlation coefficient
ICU: Intensive care unit
IQR: Interquartile range
KDPI: Kidney donor profile index
LE: Lower extremity
LOS: Length of stay
POD: Postoperative day
QoR-15: Quality of recovery 15-item survey
RF: Rectus femoris
SD: Standard deviation
SNF: Skilled nursing facility

## References

[1] Bello AK, Okpechi IG, Levin A, Ye F, Damster S, Arruebo S, Donner JA, Caskey FJ, Cho Y, Davids MR, et al. An update on the global disparities in kidney disease burden and care across world countries and regions. Lancet Glob Health. 2024;12(3):e382–95. 38365413 10.1016/S2214-109X(23)00570-3

[2] McAdams-Demarco MA, Grams ME, King E, Desai NM, Segev DL. Sequelae of early hospital readmission after kidney transplantation. Am J Transplant. 2014;14(2):397–403. 24447652 10.1111/ajt.12563 PMC3998748

[3] Sheshadri A, Elia JR, Garcia G, Abrams G, Adey DB, Lai JC, Sudore RL. Barriers and facilitators to exercise in older adults awaiting kidney transplantation and their care partners. Kidney Med. 2024;6(3): 100779. 38419789 10.1016/j.xkme.2023.100779 PMC10900112

[4] Knoedler S, Schliermann R, Knoedler L, Wu M, Hansen FJ, Matar DY, Obed D, Vervoort D, Haug V, Hundeshagen G, et al. Impact of sarcopenia on outcomes in surgical patients: a systematic review and meta-analysis. Int J Surg. 2023;109(12):4238–62. 37696253 10.1097/JS9.0000000000000688 PMC10720826

[5] Wong L, Kent AB, Lee D, Roberts MA, McMahon LP. Low muscle mass and early hospital readmission post-kidney transplantation. Int Urol Nephrol. 2022. 10.1007/s11255-021-03085-1. 10.1007/s11255-021-03085-1 35028810

[6] Deliege PG, Braconnier A, Chaix F, Renard Y, Petrache A, Guyot-Colosio C, Kazes I, Mokri L, Barbe C, Rieu P. Skeletal muscle index as a prognostic marker for kidney transplantation in older patients. J Ren Nutr. 2021;31(3):286–95. 33139208 10.1053/j.jrn.2020.08.014

[7] Tabourin T, Pinar U, Cassagnes L, Boirie Y, Heng AE, Guandalino M, Guy L. The role of CT-scan assessment of muscle mass in predicting postoperative surgical complications after renal transplantation. Int Urol Nephrol. 2022;54(3):517–23. 34897571 10.1007/s11255-021-03089-x

[8] Sabatino A, Cuppari L, Stenvinkel P, Lindholm B, Avesani CM. Sarcopenia in chronic kidney disease: what have we learned so far? J Nephrol. 2021;34(4):1347–72. 32876940 10.1007/s40620-020-00840-y PMC8357704

[9] Sabatino A, D’Alessandro C, Regolisti G, di Mario F, Guglielmi G, Bazzocchi A, Fiaccadori E. Muscle mass assessment in renal disease: the role of imaging techniques. Quant Imaging Med Surg. 2020;10(8):1672–86. 32742960 10.21037/qims.2020.03.05 PMC7378093

[10] Nijholt W, Scafoglieri A, Jager-Wittenaar H, Hobbelen JSM, van der Schans CP. The reliability and validity of ultrasound to quantify muscles in older adults: a systematic review. J Cachexia Sarcopenia Muscle. 2017;8(5):702–12. 28703496 10.1002/jcsm.12210 PMC5659048

[11] Mueller N, Murthy S, Tainter CR, Lee J, Riddell K, Fintelmann FJ, Grabitz SD, Timm FP, Levi B, Kurth T, et al. Can sarcopenia quantified by ultrasound of the rectus femoris muscle predict adverse outcome of surgical intensive care unit patients as well as frailty? A prospective, observational cohort study. Ann Surg. 2016;264(6):1116–24. 26655919 10.1097/SLA.0000000000001546 PMC4907876

[12] Canales C, Mazor E, Coy H, Grogan TR, Duval V, Raman S, Cannesson M, Singh SP. Preoperative point-of-care ultrasound to identify frailty and predict postoperative outcomes: a diagnostic accuracy study. Anesthesiology. 2022;136(2):268–78. 34851395 10.1097/ALN.0000000000004064 PMC9843825

[13] Yang Q, Zhang C, Zhang Z, Su B. Muscle ultrasound to diagnose sarcopenia in chronic kidney disease: a systematic review and bayesian bivariate meta-analysis. BMC Nephrol. 2024;25(1):12. 38178026 10.1186/s12882-023-03445-2 PMC10768384

[14] Gould DW, Watson EL, Wilkinson TJ, Wormleighton J, Xenophontos S, Viana JL, Smith AC. Ultrasound assessment of muscle mass in response to exercise training in chronic kidney disease: a comparison with MRI. J Cachexia Sarcopenia Muscle. 2019;10(4):748–55. 31054219 10.1002/jcsm.12429 PMC6711420

[15] Matsuzawa R, Yamamoto S, Suzuki Y, Imamura K, Harada M, Matsunaga A, Tamaki A, Fukui T, Shimokado K. The clinical applicability of ultrasound technique for diagnosis of sarcopenia in hemodialysis patients. Clin Nutr. 2021;40(3):1161–7. 32798065 10.1016/j.clnu.2020.07.025

[16] Guimaraes J, Araujo AM, Santos F, Nunes CS, Casal M. Living-donor and deceased-donor renal transplantation: differences in early outcome–a single-center experience. Transplant Proc. 2015;47(4):958–62. 26036494 10.1016/j.transproceed.2015.03.008

[17] Kolodzie K, Cakmakkaya OS, Boparai ES, Tavakol M, Feiner JR, Kim MO, Newman TB, Niemann CU. Perioperative normal saline administration and delayed graft function in patients undergoing kidney transplantation: a retrospective cohort study. Anesthesiology. 2021;135(4):621–32. 34265037 10.1097/ALN.0000000000003887

[18] von Elm E, Altman DG, Egger M, Pocock SJ, Gotzsche PC, Vandenbroucke JP, Initiative S. The Strengthening the Reporting of Observational Studies in Epidemiology (STROBE) statement: guidelines for reporting observational studies. Lancet. 2007;370(9596):1453–7. 18064739 10.1016/S0140-6736(07)61602-X

[19] Bell M, Eriksson LI, Svensson T, Hallqvist L, Granath F, Reilly J, Myles PS. Days at home after surgery: an integrated and efficient outcome measure for clinical trials and quality assurance. EClinicalMedicine. 2019;11:18–26. 31317130 10.1016/j.eclinm.2019.04.011 PMC6610780

[20] Jerath A, Austin PC, Wijeysundera DN. Days alive and out of hospital: validation of a patient-centered outcome for perioperative medicine. Anesthesiology. 2019;131(1):84–93. 31094760 10.1097/ALN.0000000000002701

[21] Myles PS, Shulman MA, Heritier S, Wallace S, McIlroy DR, McCluskey S, Sillar I, Forbes A. Validation of days at home as an outcome measure after surgery: a prospective cohort study in Australia. BMJ Open. 2017;7(8): e015828. 10.1136/bmjopen-2017-015828 PMC5629653 28821518

[22] Clavien PA, Barkun J, de Oliveira ML, Vauthey JN, Dindo D, Schulick RD, de Santibanes E, Pekolj J, Slankamenac K, Bassi C, et al. The clavien-dindo classification of surgical complications: five-year experience. Ann Surg. 2009;250(2):187–96. 19638912 10.1097/SLA.0b013e3181b13ca2

[23] Grochowiecki T, Madej K, Galazka Z, Jakimowicz T, Jedrasik M, Swiercz P, Lukawski K, Paczek L, Durlik M, Nazarewski S, et al. Usefulness of modified Dindo-Clavien scale to evaluate the correlation between the severity of surgical complications and complications related to the renal and pancreatic grafts after simultaneous kidney and pancreas transplantation. Transplant Proc. 2016;48(5):1677–80. 27496470 10.1016/j.transproceed.2016.01.091

[24] Mendez NV, Raveh Y, Livingstone JJ, Ciancio G, Guerra G, Burke Iii GW, Shatz VB, Souki FG, Chen LJ, Morsi M, et al. Perioperative risk factors associated with delayed graft function following deceased donor kidney transplantation: A retrospective, single center study. World J Transplant. 2021;11(4):114–28. 33954089 10.5500/wjt.v11.i4.114 PMC8058644

[25] Barr ML, Belghiti J, Villamil FG, Pomfret EA, Sutherland DS, Gruessner RW, Langnas AN, Delmonico FL. A report of the Vancouver forum on the care of the live organ donor: lung, liver, pancreas, and intestine data and medical guidelines. Transplantation. 2006;81(10):1373–85. 16732172 10.1097/01.tp.0000216825.56841.cd

[26] Stark PA, Myles PS, Burke JA. Development and psychometric evaluation of a postoperative quality of recovery score: the QoR-15. Anesthesiology. 2013;118(6):1332–40. 23411725 10.1097/ALN.0b013e318289b84b

[27] Hammond K, Mampilly J, Laghi FA, Goyal A, Collins EG, McBurney C, Jubran A, Tobin MJ. Validity and reliability of rectus femoris ultrasound measurements: comparison of curved-array and linear-array transducers. J Rehabil Res Dev. 2014;51(7):1155–64. 25437305 10.1682/JRRD.2013.08.0187

[28] Myles PS, Myles DB, Galagher W, Chew C, MacDonald N, Dennis A. Minimal clinically important difference for three quality of recovery scales. Anesthesiology. 2016;125(1):39–45. 27159009 10.1097/ALN.0000000000001158

[29] Myles PS, Myles DB. An updated minimal clinically important difference for the QoR-15 scale. Anesthesiology. 2021;135(5):934–5. 34543410 10.1097/ALN.0000000000003977

[30] Druckmann I, Yashar H, Schwartz D, Schwartz IF, Goykhman Y, Kliuk Ben-Bassat O, Baruch R, Tzadok R, Shashar M, Cohen-Hagai K, et al. Presence of sarcopenia before kidney transplantation is associated with poor outcomes. Am J Nephrol. 2022;53(6):427–34. 35584614 10.1159/000524774 PMC9393828

[31] Kim HJ, Hong N, Kim HW, Yang J, Kim BS, Huh KH, Kim MS, Lee J. Low skeletal muscle mass is associated with mortality in kidney transplant recipients. Am J Transplant. 2023;23(2):239–47. 36695681 10.1016/j.ajt.2022.11.016

[32] Karakizlis H, Trudel N, Brose A, Reinisch A, Reichert M, Hecker A, Bender F, Askevold I, Rainer L, Weimer R, et al. Sarcopenia of kidney transplant recipients as a predictive marker for reduced graft function and graft survival after kidney transplantation. Langenbecks Arch Surg. 2023;408(1):103. 36826595 10.1007/s00423-023-02836-1 PMC9958183

[33] Lentine KL, Smith JM, Hart A, Miller J, Skeans MA, Larkin L, Robinson A, Gauntt K, Israni AK, Hirose R, et al. OPTN/SRTR 2020 annual data report: kidney. Am J Transplant. 2022;22(Suppl 2):21–136. 35266618 10.1111/ajt.16982

[34] Kobashigawa J, Dadhania D, Bhorade S, Adey D, Berger J, Bhat G, Budev M, Duarte-Rojo A, Dunn M, Hall S, et al. Report from the American Society of Transplantation on frailty in solid organ transplantation. Am J Transplant. 2019;19(4):984–94. 30506632 10.1111/ajt.15198 PMC6433498

[35] McAdams-DeMarco MA, Ying H, Van Pilsum Rasmussen S, Schrack J, Haugen CE, Chu NM, Gonzalez Fernandez M, Desai N, Walston JD, Segev DL. Prehabilitation prior to kidney transplantation: results from a pilot study. Clin Transplant. 2019;33(1): e13450. 30462375 10.1111/ctr.13450 PMC6342659

[36] Mayer KP, Teixeira JP, Gonzalez-Seguel F, Tran VQ, Gross JM, Horikawa-Strakovsky A, Pal CA, Shareef ZT, Puffer Israel H, Wen Y, et al. Acute skeletal muscle wasting in patients with acute kidney injury requiring continuous kidney replacement therapy: a prospective multicenter study. J Crit Care. 2025;89: 155142. 40513259 10.1016/j.jcrc.2025.155142 PMC12303733

[37] Teixeira JP, Mayer KP, Griffin BR, George N, Jenkins N, Pal CA, Gonzalez-Seguel F, Neyra JA. Intensive care unit-acquired weakness in patients with acute kidney injury: a contemporary review. Am J Kidney Dis. 2023;81(3):336–51. 36332719 10.1053/j.ajkd.2022.08.028 PMC9974577

