# Supplementary material for: Ultrasound-based assessment of muscle mass is associated with early recovery after kidney transplant: a prospective single-center study
Source: BMC Anesthesiol. 2025 Aug 26;25:424. doi: 10.1186/s12871-025-03288-4 (PMC12379526; doi:10.1186/s12871-025-03288-4)
Supplement: Supplementary file 1 — Supplementary Material 1. [file 12871_2025_3288_MOESM1_ESM.pdf]

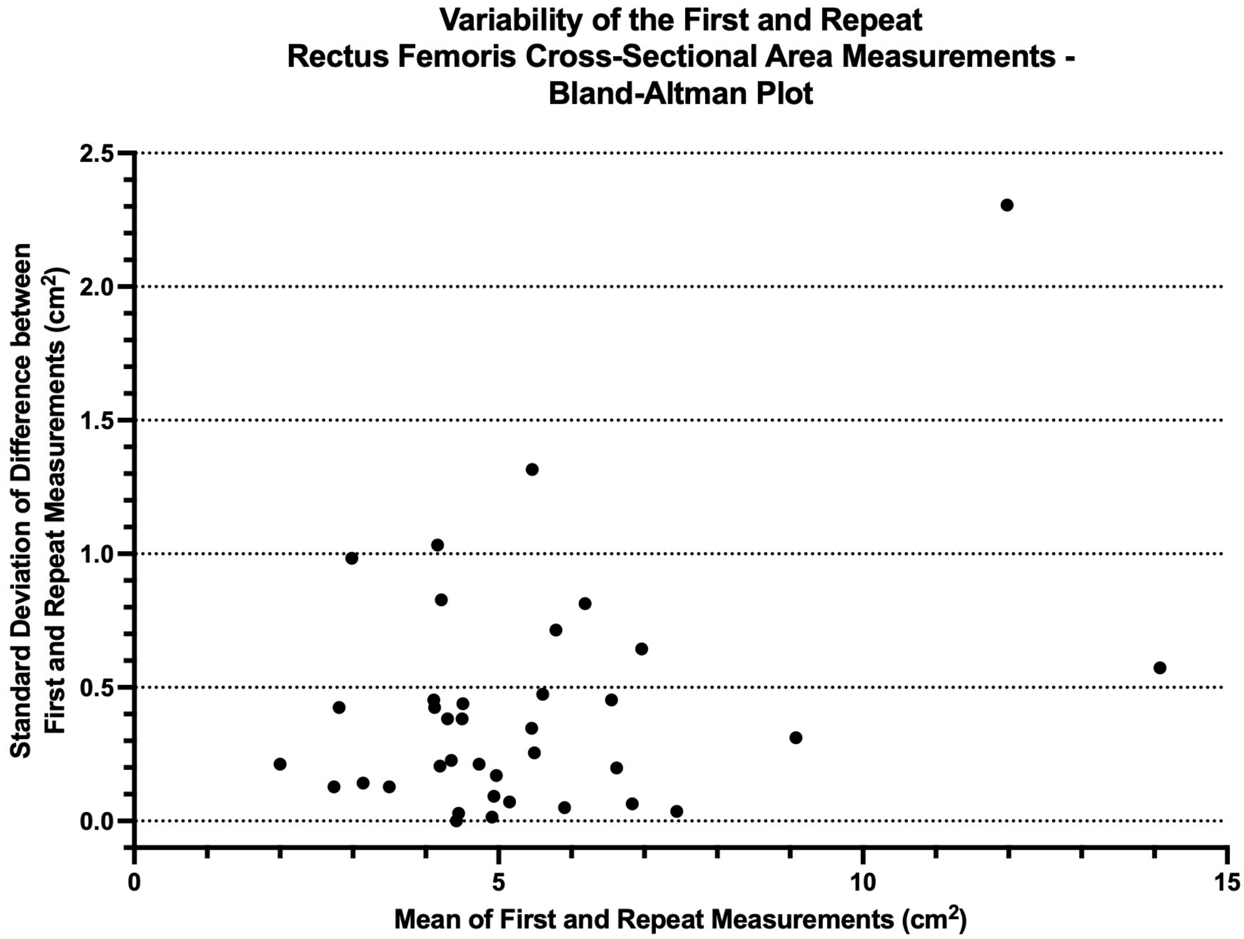

Supplemental Digital Content 1. Bland-Altman plot evaluating the within subject standard deviation over the range of within subject mean values of the first and repeat rectus femoris muscle cross-sectional areas measured by ultrasound.
