# Supplementary material for: Ultrasound-based assessment of muscle mass is associated with early recovery after kidney transplant: a prospective single-center study
Source: BMC Anesthesiol. 2025 Aug 26;25:424. doi: 10.1186/s12871-025-03288-4 (PMC12379526; doi:10.1186/s12871-025-03288-4)
Supplement: Supplementary file 2 — Supplementary Material 2. [file 12871_2025_3288_MOESM2_ESM.pdf]

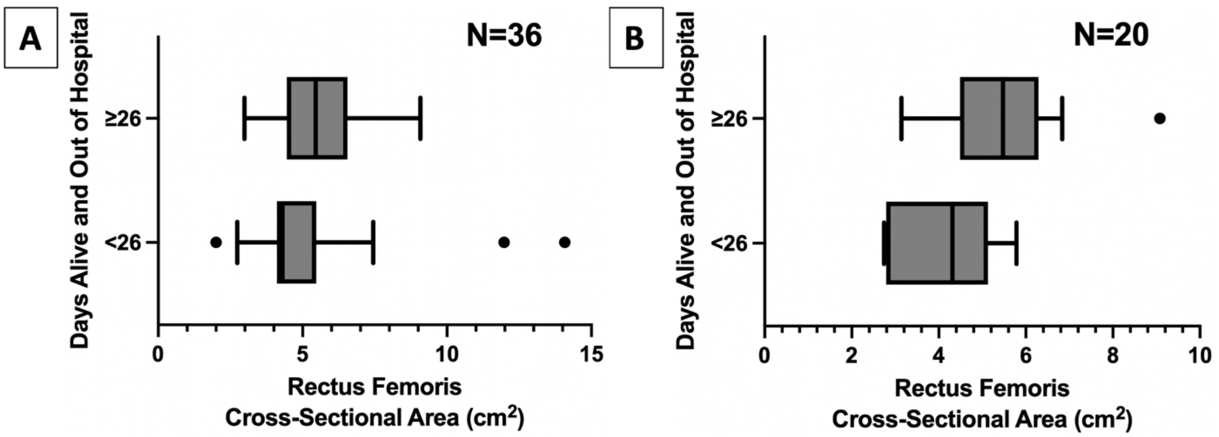

Supplemental digital content 2. A sensitivity analysis was conducted to assess the robustness as well as to identify influential factors related to the finding that ultrasound-measured preoperative muscle mass is associated with the number of days alive and out of the hospital within the 30 days (DAH_{30}) after surgery. For the sake of this sensitivity analysis, we instead dichotomized the cohort into those patients who experienced 26 or more versus those who experienced fewer than 26 DAH_{30}. When doing so, the median RF CSA for those with <26 days was 4.3 cm^2 (IQR 4.12 to 5.15) compared with 5.46 cm^2 (IQR 4.45 to 6.55) for those with 26 or more DAH_{30}. Non-parametric testing indicates no statistically significant difference (p=0.114). In looking at the boxplot (Figure A above), we surmise that the two extreme outliers largely drive this non-significant result. When the same analysis is repeated with exclusion of those two data points, it results in a statistically significant difference (p=0.015). Both outliers are patients who developed delayed graft function (DGF) and required a second surgical intervention during the primary admission. While this study is not large enough to investigate this finding in detail, we believe that the data demonstrates a signal allowing for hypothesis generation that may inform and encourage future larger studies. Given the nature of DGF, it is a complication that is unlikely to be related to preoperative frailty, and in fact, if we run the same analysis in only the 20 patients who did not develop DGF, we see a very statistically significant difference (p=0.023) (Figure B above).
